# Effects of Long-Term Fertilizer Practices on Rhizosphere Soil Autotrophic CO_2_-Fixing Bacteria under Double Rice Ecosystem in Southern China

**DOI:** 10.4014/jmb.2205.05055

**Published:** 2022-09-26

**Authors:** Haiming Tang, Li Wen, Lihong Shi, Chao Li, Kaikai Cheng, Weiyan Li, Xiaoping Xiao

**Affiliations:** Hunan Soil and Fertilizer Institute, Changsha 410125, P.R. China

**Keywords:** Rice, fertilizer treatment, crop residue, soil autotrophic bacteria, paddy field

## Abstract

Soil autotrophic bacterial communities play a significant role in the soil carbon (**C**) cycle in paddy fields, but little is known about how rhizosphere soil microorganisms respond to different long-term (35 years) fertilization practices under double rice cropping ecosystems in southern China. Here, we investigated the variation characteristics of rhizosphere soil RubisCO gene *cbbL* in the double rice ecosystems of in southern China where such fertilization practices are used. For this experiment we set up the following fertilizer regime: without any fertilizer input as a control (CK), inorganic fertilizer (MF), straw returning (RF), and organic and inorganic fertilizer (OM). We found that abundances of *cbbL*, 16S rRNA genes and RubisCO activity in rhizosphere soil with OM, RF and MF treatments were significantly higher than that of CK treatment. The abundances of *cbbL* and 16S rRNA genes in rhizosphere soil with OM treatment were 5.46 and 3.64 times higher than that of CK treatment, respectively. Rhizosphere soil RubisCO activity with OM and RF treatments increased by 50.56 and 45.22%, compared to CK treatment. Shannon and Chao1 indices for rhizosphere soil *cbbL* libraries with RF and OM treatments increased by 44.28, 28.56, 29.60, and 23.13% compared to CK treatment. Rhizosphere soil *cbbL* sequences with MF, RF and OM treatments mainly belonged to *Variovorax paradoxus*, uncultured proteobacterium, *Ralstonia pickettii*, *Thermononospora curvata*, and *Azoarcus* sp.KH33C. Meanwhile, *cbbL*-carrying bacterial composition was obviously influenced by soil bulk density, rhizosphere soil dissolved organic C, soil organic C, and microbial biomass C contents. Fertilizer practices were the principal factor influencing rhizosphere soil *cbbL*-carrying bacterial communities. These results showed that rhizosphere soil autotrophic bacterial communities were significantly changed under conditions of different long-term fertilization practices Therefore, increasing rhizosphere soil autotrophic bacteria community with crop residue and organic manure practices was found to be beneficial for management of double rice ecosystems in southern China.

## Introduction

It is generally believed that soil autotrophic bacteria typically exist in agricultural soils [1-3], and play a vital role in helping to regulate the carbon cycle while promoting net uptake of atmospheric carbon dioxide (CO_2_) [4, 5]. In the previous studies, results have demonstrated the incorporation of CO_2_ into soil microbial biomass at rate of 0.01–0.10 g C/m^2^/day with soil autotrophic bacteria [4, 6]. Soil autotrophic bacterial composition and diversity were obviously influenced by applying different field practices, such as cropping, fertilization management, crop straw, tillage, etc. [1, 7]. For these reasons, there is a need to explore the impact of different fertilization practices on rhizosphere soil autotrophic bacteria composition and diversity in paddy fields.

Previous results have shown that soil autotrophic microbes were significantly influenced by different fertilization management practices, such as changing soil physical and biogeochemistry characteristics, and thus, soil autotrophic bacterial community and diversity were also altered [7, 8]. In particular, biomass, activity, abundances and composition of soil autotrophic bacteria were changed by using different fertilization practices [9]. Yuan *et al*. (2012) [10] found that soil *cbbL*-containing bacterial community was dominated by facultative autotrophic bacteria, and RubisCO activity in paddy field was significantly enhanced under application of crop straw and organic manure conditions. Increasing CO_2_ sequestration and decreasing greenhouse gas (N_2_O and CH_4_) emissions from agricultural soils through application of crop straw and organic manure conditions were found to be beneficial for crop management [11]. Some studies investigated the mechanisms of denitrification and methanogenesis based on investigation of 16S rRNA and specific functional genes [7, 12]. However, soil microbial CO_2_ assimilation ecological processes in paddy fields by using molecular method still need to be explored.

At present, use of *cbbL* gene is considered effective for gathering information about soil autotrophic bacterial communities as this gene thought to be a functional marker for analyzing soil autotrophic bacterial composition due to its crucial involvement in the Calvin cycle [10, 13]. Previous studies investigated soil *cbbL*-carrying bacterial community and diversity by using molecular biology techniques (*e.g.*, RFLP, TRFLP, PLFA and RT–PCR)[6, 7, 14]. According to these approaches, it has been reported that abundances of 16S rRNA and *cbbL* genes were significantly enhanced by applying straw or biochar treatments, compared to without straw or biochar input treatments [15]. However, rhizosphere soil *cbbL*-carrying bacterial community and diversity in paddy field in response to different fertilization practices still need further investigation.

Rice is the major grain crop in Asia [16], and double rice cropping (early and late rice planted within a single year) is a common planting system in southern China. The application of organic and inorganic fertilizers is seen as a beneficial practice for enhancing soil physical and chemical properties in paddy fields. Previous studies reported that soil bulk density, soil pH, and soil organic carbon (SOC) content were obviously altered by different fertilization practices [17, 18], which affect soil C sequestration and microbial properties in paddy fields. However, there is also a need to investigate the response of soil C sequestration microbial properties according to different fertilization practices under double rice ecosystem in southern China. Therefore, we set up different fertilizer regimes in paddy fields under double rice ecosystem south China. Our objective in this experiment was as follows: (1) to calculate changes of rhizosphere soil autotrophic bacterial composition and activity by different fertilization practices; (2) to analyze the relationship between soil physiochemical characteristics and soil *cbbL*-carrying bacterial community, as well as RubisCO activity under double rice ecosystem.

## Materials and Methods

### Field Experiment Site

The fertilizer experiment was located under a double-cropped rice field near Ningxiang (28°07′ N, 112°18′ E), Hunan Province, in southern China. The related information about climatic characteristics during this field experiment, cropping system, soil chemical properties at plough layer in paddy field at the beginning of fertilizer experiment (1986) was as described by Tang *et al*. (2018) [18].

### Experiment Design

This experiment applied the following fertilizer regime: without any fertilizer input as a control (CK), inorganic fertilizer (MF), straw returning (RF), and organic and inorganic fertilizer (OM). Also utilized was a randomized block design for each fertilizer treatment in paddy field with three replications, and the area of each treatment was 66.7 m^2^ (10.0 × 6.67 m). We kept the same levels of nitrogen (N), phosphorus pentoxide (P_2_O_5_) and potassium oxide (K_2_O) with OM, RF and MF treatments during the whole growth stage of early rice and late rice, respectively. Other related and more detailed information about the fertilization practices (applied with the kinds and date of fertilizer, total amount of fertilizer) and other field management methods (rice varieties, transplanting density, irrigation pattern) were as as described by Tang *et al*. (2018) [18].

### Soil Sample Collection

Rhizosphere soil samples were collected by randomly taking 20 rice plants from each fertilizer treatment, at maturity stage of late rice, in October 2020. Therefore, three composite soil samples with each fertilizer treatment were collected at sampling time, and these soil samples were divided into two parts. One part of the soil sample was stored at 4°C for investigation of soil chemical characteristics; the other part the of soil sample was kept at −20°C for molecular biological analysis.

### Soil Physiochemical Characteristics Analysis

Soil bulk density at plough layer in paddy field was measured according to the method as introduced by Blake and Hartge (1986) [19]. Soil pH, soil organic carbon, total nitrogen, available phosphorus and available potassium contents were measured based on the method of Kjeldahl (1996) [20]. Soil dissolved organic carbon content was analyzed based on the method as described by Jones and Willett (2006) [21]. Soil microbial biomass carbon content was measured by using the fumigation–extraction method introduced by Wu *et al*. (1990) [22]. Meanwhile, soil RubisCO activity was measured based on the method of Ezaki *et al*. (1999) [23].

### Soil DNA Extraction and Illumina High-Throughput Sequencing

Soil microbial DNA was collected from soil sample (0.4 g) by using the Quick Soil Isolation Kit (HuaYueYang Biotechnology Co., Ltd., China). Soil *cbbL* gene was amplified with primers V2r (5′–barcode–GCCTTC[C/G]AGCTTGCC[/G]ACC[G/A]C-3′) and K2f (5′–barcode–ACCA[C/T]CAAGCC[G/C]AAGCT[C/G]GG–3′)[7] by using a thermocycler (ABI Gene Amp 9700, Axygen Biosciences, USA). Related and more detailed conditions on the polymerase chain reaction (PCR) were as described by Yuan *et al*. (2013) [7]. Finally, soil PCR products were sent to OE-Biotech Company (China) for Illumina high-throughput sequencing.

### Soil Bacterial *cbbL* and 16S rRNA Genes

Soil bacterial *cbbL* gene quantification was done by using real–time quantitative PCR with the same primers as introduced above for *cbbL*, and soil 16S rRNA gene was amplified with primers Eub518 (5′–ATTACCGCGGCTGCT GG–3′) and Eub338 (5′–ACTCCTACGGGAGGCA GCAG–3′) [24]. Related, more detailed conditions about the PCR for soil *cbbL* and 16S rRNA genes abundances were as described by Lu *et al*. (2019) [24]. Soil *cbbL* gene abundance (copies/g soil) was analyzed according to soil DNA template (5 ng/μl) to each gram of soil (ng/g soil).

### High-Throughput Sequencing Data Analysis

Raw fastq files were quality checked by using Trimmomatic (Version 3.29) and merged by using FLASH (v1.2.7) software, respectively, according to the following standards: (i) These reads were interrupted an average quality score < 20 over 50 bp sliding window. (ii) Sequences were merged based on their overlap (> 10 bp), mismatching below 2 bp was allowed during this step. (iii) Sequences in all soil samples were segregated based on the primers and barcodes, and reads containing ambiguous bases were deleted. The peak areas of terminal restriction fragments with difference of ±1 bp were added and regarded as fragments of the *cbbL* gene operational taxonomic units (OTUs) in the sample. Soil alpha diversity was analyzed by using Chao1, and diversity was analyzed by using Shannon index.

OTUs were clustered with 97% sequence identity by using UPARSE software (Version 7.1), and chimeric filtering was conducted at the same time. The classification of each *cbbL* sequence was annotated by the Nucleotide database in the National Center for Biotechnology Information (NCBI). Family and genus were designated according to amino acid sequence similarity of 90% and 95%, respectively. All high-throughput sequencing data with soil sample were submitted to NCBI Sequence Read Archive (SRA) under SRA accession number SRP142452.

### Statistical Analysis

Data for each investigated item in all fertilizer treatments were analyzed by using one-way analysis of variance (ANOVA) (*p*-value 0.05). The results for each item were shown as mean ± standard error. Statistical analysis was done with SAS software (Version 9.3) [25]. The relationship between soil physiochemical characteristics and soil microbial composition was analyzed with canonical correspondence analysis (CCA). Meanwhile, soil microbial community change at OTU level was evaluated with principal component analyses (PCA). The correlation test, CCA and PCA analyses were conducted with ‘vegan’ package (Version 3.20).

## Results

### Abundance of Rhizosphere Soil Bacterial *cbbL* and 16S rRNA Genes

The results indicated that abundance of rhizosphere soil *cbbL* gene with all fertilizer treatments (MF, RF, OM and CK) ranged from 0.54 to 2.95 × 10^8^ copies/g. Therefore, abundance of rhizosphere soil *cbbL* gene with MF, RF and OM treatments increased by 2.72, 3.44, and 5.46 times higher than that of CK treatment, respectively. The results also showed that abundance of 16S rRNA gene with MF, RF, OM and CK treatments ranged from 6.69 to 24.38 × 10^9^ copies/g. Therefore, abundance of 16S rRNA gene with MF, RF and OM treatments increased by 2.06, 2.93, and 3.64 times higher than that of CK treatment, respectively (Table 1).

There were positive correlations (*p* < 0.05) between abundance of rhizosphere soil *cbbL* gene and soil dissolved organic carbon content, but there were negative correlations (*p* < 0.05) between abundance of rhizosphere soil *cbbL* gene and soil bulk density (Table 2). Meanwhile, there were positive correlations between abundance of rhizosphere soil 16S rRNA gene and soil dissolved organic carbon content, and abundance of rhizosphere soil *cbbL* gene, but the correlations were not significant (*p* > 0.05).

### Diversity of Rhizosphere Soil Bacterial *cbbL* and 16S rRNA Genes

**Fig. 1:**
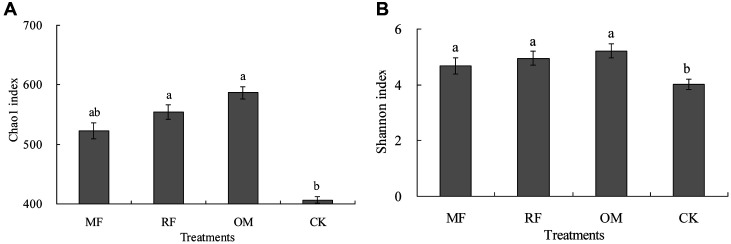
Effects of different fertilizer treatments on rhizosphere soil bacterial α-diversity for *cbbL* libraries in the double-cropping rice field. MF: chemical fertilizer alone; RF: rice straw and chemical fertilizer; OM: 30% organic manure and 70% chemical fertilizer; CK: without fertilizer input as a control. a, b represent Chao1 index and Shannon index, respectively. Different lowercase letters indicate significant difference at a *p* < 0.05 level. The same as below.

The results indicated that rhizosphere soil Chao1 and Shannon indices for *cbbL* libraries with MF, RF and OM treatments were enhanced, compared with CK treatment. Compared with CK treatment, Chao1 index with OM and RF treatments was significantly increased (*p* < 0.05). However, there was no significant (*p* > 0.05) difference in Chao1 index between OM and RF treatments (Fig. 1A). This result showed that Shannon index with MF, RF and OM treatments was significantly (*p* < 0.05) higher than that of CK treatment. However, there was no significant difference (*p* > 0.05) in Shannon index between MF, RF and OM treatments (Fig. 1B).

**Fig. 2:**
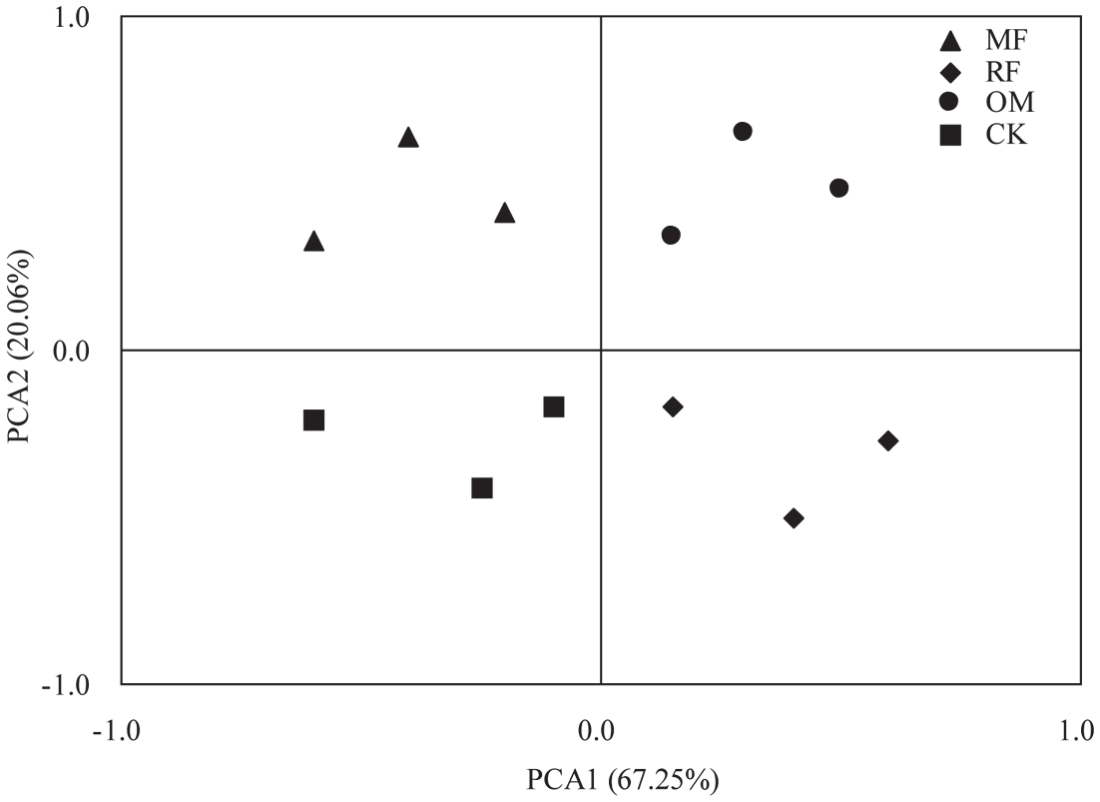
Principal component analysis (PCA) of *cbbL* library in rhizosphere soil with different fertilizer treatments. The *cbbL* distributions were based on the relative abundance of OTU. *n* = 3.

Principal component analysis (PCA) result showed that first principal component analysis (PCA1) of soil *cbbL* gene was explained 67.25%, indicating that difference in fertilizer regime was the most vital factor influencing soil *cbbL*-carrying bacteria community (Fig. 2). Our result showed that second principal component analysis (PCA2) of soil *cbbL* gene was explained 20.06%, indicating that rhizosphere was the second important factor influencing soil *cbbL*-carrying bacteria community (Fig. 2).

**Fig. 3:**
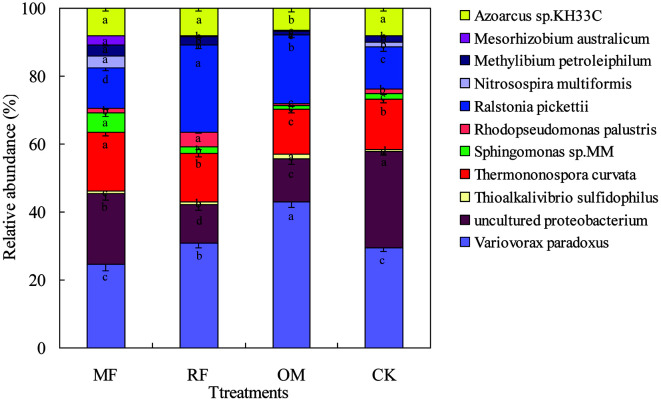
Relative abundance of *cbbL* in rhizosphere soil with different fertilizer treatments. Relative abundance of the *cbbL*-carrying bacteria in rhizosphere soil with different fertilizer treatments. Each relative abundance of bacteria was analyzed using ANOVA following standard at a 0.05 probability level. Different lowercase letters indicate significant differences at a *p* < 0.05 level.

In the present study, rhizosphere soil *cbbL*-carrying bacteria with all fertilizer treatments mainly belonged to *Actinobacteria* and *Proteobacteria*. The top 11 abundant species of rhizosphere soil *cbbL* with MF, RF, OM and CK treatments were identified. However, there were the same top five abundant species of rhizosphere soil *cbbL* with all fertilizer treatments, including *Variovorax paradoxus*, uncultured proteobacterium, *Ralstonia pickettii*, *Thermononospora curvata*, and *Azoarcus* sp.KH33C. This result showed that abundance of *V. paradoxus* with MF, RF and CK treatments was significantly decreased (*p* < 0.05), compared to OM treatment. Compared to RF, OM and CK treatments, abundance of *Sphingomonas* sp.MM and *T. curvata* with MF treatment was significantly increased (*p* < 0.05). However, the abundance of *Ralstonia pickettii* with MF, OM and CK treatments was significantly lower (*p* < 0.05) than that of RF treatment (Fig. 3).

### Rhizosphere Soil RubisCO Enzyme Activity

Rhizosphere soil RubisCO activity with all fertilizer treatments (MF, RF, OM and CK) ranged from 3.56 to 5.36 nmol CO_2_/g/min (Table 1). Rhizosphere soil RubisCO activity with MF and CK treatments was significantly lower (*p* < 0.05) than that of OM and RF treatments (Table 1). Rhizosphere soil RubisCO activity showed negative correlations (*p* < 0.05) with soil bulk density and microbial biomass carbon content, but positive correlations (*p* < 0.05) with abundance of soil *cbbL* gene (Table 2). These results indicated that RF and OM treatments were beneficial fertilizer practices for enhancing rhizosphere soil CO_2_ fixation activity under more abundance of *cbbL*-carrying bacteria and smaller soil bulk density conditions.

### Rhizosphere Soil *cbbL* Bacterial Community

**Fig. 4:**
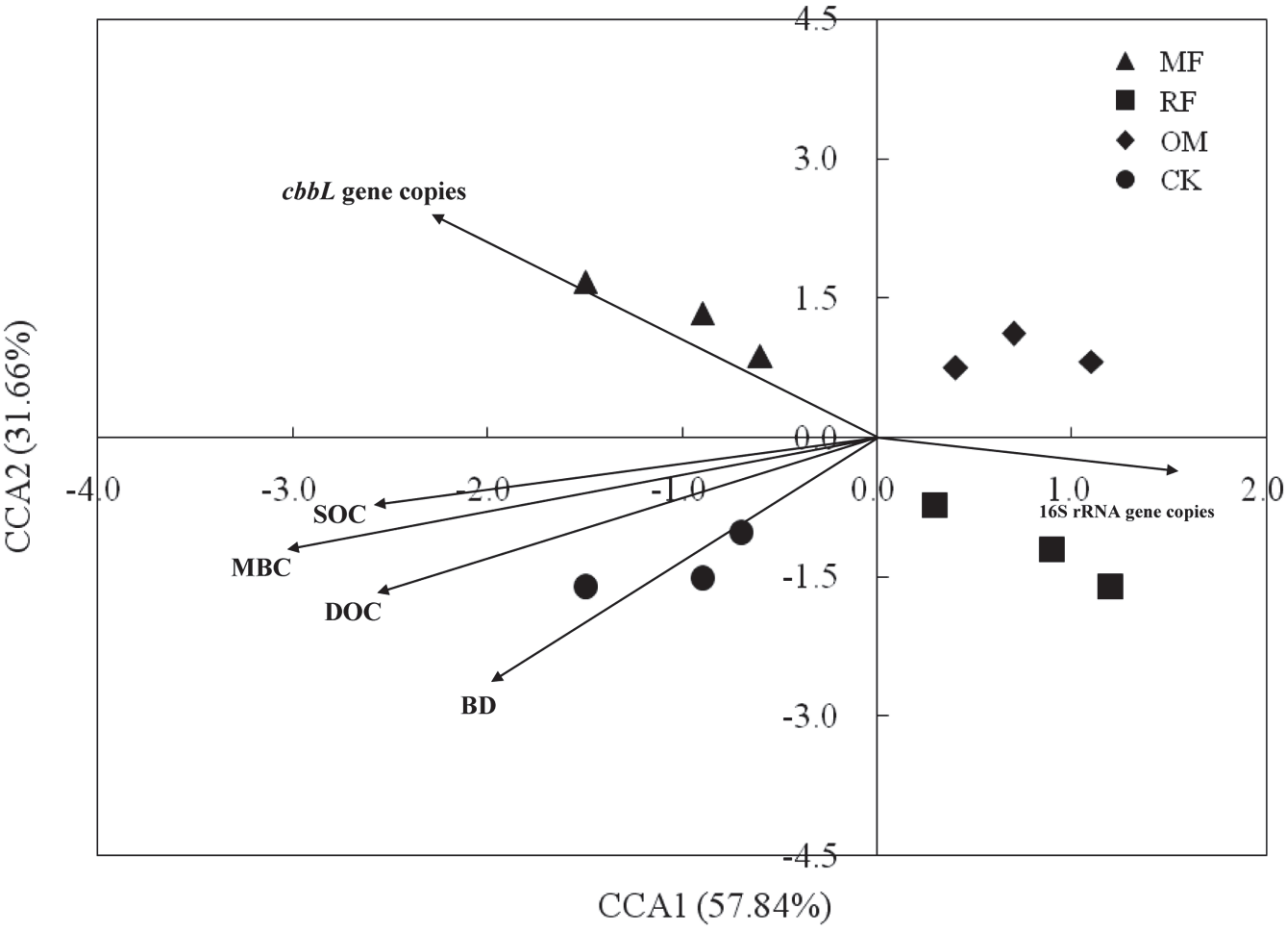
Canonical correspondence analysis (CCA) of rhizosphere soil physiochemical characteristics, *cbbL* and 16S rRNA genes copies with different fertilizer treatments. SOC: soil organic carbon; MBC: soil microbial biomass carbon; DOC: soil dissolved organic carbon; BD: soil bulk density. The direction of arrow was point coordinate axis represented the correlation. There had correlation between arrow length and investigated items.

The canonical correspondence analysis (CCA) result showed that rhizosphere soil MBC, SOC, DOC contents, soil bulk density, and *cbbL* gene copies were vital factors influencing on *cbbL*-carrying bacterial community (Fig. 4). Meanwhile, there was significant (*p* < 0.05) relationship between rhizosphere soil MBC, SOC, DOC contents and soil *cbbL*-carrying bacterial community. That is, *cbbL*-carrying bacterial communities were hugely influenced by rhizosphere soil MBC, SOC and DOC contents in paddy fields.

## Discussion

In previous studies, soil bacterial community were positively affected by organic manure and crop straw practices, resulting in increased soil bacterial abundance and activity, compared with inorganic fertilizer management [3, 7, 10, 15]. In this study, our results demonstrated that rhizosphere soil *cbbL*-carrying bacteria abundance and soil carbon dioxide (CO_2_) fixation activities were promoted with OM and RF treatments. Meanwhile, our own previous study results indicated that rhizosphere soil organic carbon (SOC) and soil microbial biomass carbon (SMBC) contents were also enhanced with OM and RF treatments [26], which suggested that it was an effective practice to provide a suitable soil ecology condition and nutrient content in the root region for soil microorganism growth under the application of crop straw or organic manure conditions. Therefore, changes of soil microbial community were mainly attributed to SOC content. At the same time, our results indicated that rhizosphere soil *cbbL* gene abundance and soil RubisCO activity were enhanced with RF and OM treatments, compared to CK treatment. This was attributed to promotion of SOC, SMBC, dissolved organic carbon (DOC) and soil total nitrogen (N) content by OM and RF treatments. On the other hand, methane oxidation activity was reduced in higher rhizosphere soil mineral N content condition [11], and this effect could increase soil autotrophic CO_2_ fixation activity. Therefore, RF and OM treatments were beneficial fertilizer practices for increasing growth of rhizosphere soil *cbbL*-carrying bacteria under higher soil DOC content and lower soil bulk density conditions. Meanwhile, this result showed that rhizosphere soil *cbbL* gene abundance and soil RubisCO activity with OM treatment were more than that of RF and MF treatments, which indicated that soil quality and fertility in paddy field were enhanced with organic manure practice resulted in soil porosity decrease and soil bulk density increase [15]. Meanwhile, soil gas diffusivity was significantly increased with organic manure input practice, which implied more available CO_2_ for *cbbL*-carrying bacteria [10]. As a result, it was found beneficial to increase soil bacteria activity under smaller soil bulk density condition with organic manure input practice, including soil autotrophic bacteria, endoglucanases, cellobiohydrolases, β-glucosidases, and fungal and actinobacterial cellulolytic and nitrogenase activities [27, 28], all of which enhanced soil ecology condition, carbon substrate, and soil nutrient content [7, 15].

Previous studies results showed that soil *cbbL*-carrying taxa belonged to *Alcaligenes utrophus*, *Ralstonia eutropha*, *Thiobacillus denitrificans*, *Nitrobacter vulgaris* and *Nitrobacter winogradskyi* [7, 10]. In the present study, we found that most soil autotrophic CO_2_-fixing bacteria belonged to *Proteobacteria*, while some belonged to *Actinobacteria*. Our results also demonstrated that dominant *cbbL* gene sequences with all fertilizer practices were related to *V. paradoxus*, uncultured proteobacterium, *R. pickettii*, *T. curvata*, and *Azoarcus* sp.KH33C, which were consistent with the previous results [10, 29]. These abundant species were closely attached to those previously found taxa, with the reason mainly attributed to the fact that there are more available nutrients in organic manure and crop straw soils, compared to without any fertilizer input soil [10]. Therefore, the appearance of organic manure and crop straw related to *cbbL*-carrying bacteria was induced by the presence of these soil nutrient contents. Shannon index for *cbbL* libraries with OM and RF treatments showed significantly increased soil autotrophic microorganisms, which were promoted by the process of soil autotrophic CO_2_-fixing. At the same time, these results indicated that relative abundance of *V. paradoxus* with OM and RF treatments was significantly increased, suggesting that *V. paradoxus* was usually in wide existence among soil mesophilic bacteria and obligate heterotrophic bacteria under organic manure and crop straw conditions. However, the relative abundances of *Mesorhizobium australicum*, *Sphingomonas* sp.MM and *T. curvata* in rhizosphere soil with inorganic fertilizer practice were increased, and these patterns were also reported in the previous study [10].

The present study demonstrated that rhizosphere soil *cbbL* gene abundance was positively correlated with soil chemical characteristics (soil pH, SOC, DOC, MBC, soil total N, soil available P, soil available K contents), while being negatively correlated with soil bulk density. Some results found that soil pH and other soil physicochemical characteristics play a vital role in limiting or co-limiting soil autotrophic bacteria growth [15]. Our results also suggested that soil pH and soil physicochemical characteristics provide more available nutrient for soil microbes to multiply [6, 30], suggesting that growth of soil autotrophic microbes was significantly associated with those soil physicochemical properties. These results were in accordance with Selesi *et al*. (2007) [31], who found that copy numbers of *cbbL* gene were positively correlated with soil DOC content and negatively correlated with soil bulk density. The observed correlations were related to the activities of soil autotrophic CO_2_-fixing bacteria and cellulolytic microbes being stimulated under soil DOC content conditions [27, 32]. Meanwhile, the CCA result also found that soil bulk density, MBC content, abundance of *cbbL*, and 16S rRNA genes were vital triggers for changing soil *cbbL*-carrying bacterial community (Fig. 4), indicating that it was beneficial for promoting soil autotrophic CO_2_ fixation under a lower soil bulk density environment [33]. Therefore, our results demonstrated that lower soil bulk density, more soil pH, DOC, SOC, and MBC contents were the important factors influencing rhizosphere soil autotrophic microorganism community.

## Figures and Tables

**Table 1:** Abundance of rhizosphere soil *cbbL* and 16S rRNA genes, RubisCO activity with different fertilization practices under double rice ecosystem.

Genes	Treatments			

	MF	RF	OM	CK
*cbbL* abundance (×10^8^ copies/g)	1.47 ± 0.06c	1.86 ± 0.07b	2.95 ± 0.10a	0.54 ± 0.03d
Bacterial abundance (×10^9^ copies/g)	13.72 ± 0.68c	19.63 ± 0.97b	24.38 ± 1.05a	6.69 ± 0.33d
RubisCO activity (nmol CO_2_/g/min)	4.27 ± 0.16b	5.17 ± 0.21a	5.36 ± 0.22a	3.56 ± 0.16c

MF: inorganic fertilizer; RF: straw returning; OM: organic and inorganic fertilizer; CK: without any fertilizer input as a control.

Values expressed as mean ± standard error.

Different lower case letters indicate significant difference among fertilizer treatments at *p* < 0.05.

**Table 2:** Correlation between abundance of soil *cbbL*, 16S rRNA genes, RubisCO activity, and soil physiochemical characteristics.

	pH	Total N	Available P	Available K	SOC	DOC	BD	MBC	Abundance of *cbbL* gene	Abundance of 16S rRNA gene
Abundance of *cbbL* gene	0.172	-0.365	0.306	-0.385	-0.363	0.836*	-0.803*	-0.431	—	—
Abundance of 16S rRNA gene	-0.462	-0.447	-0.103	-0.116	-0.407	0.539	-0.507	-0.336	0.584	—
RubisCO activity	-0.135	-0.508	-0.107	-0.463	-0.462	0.584	-0.824*	-0.836*	0.841*	0.603

(*) indicated significant difference at 0.05 level.

SOC: soil organic C; DOC: dissolved organic C; MBC: microbial biomass C; BD: soil bulk density.
